# Factors associated with reported need of weight loss support among adults with overweight or obesity: results from a cross-sectional population study in 2022 in Sweden

**DOI:** 10.1017/S1368980024002039

**Published:** 2024-11-12

**Authors:** Anu Molarius, Jan Karlsson

**Affiliations:** 1 Centre for Clinical Research, Region Värmland, Karlstad 651 85, Sweden; 2 Department of Public Health Sciences, Karlstad University, Karlstad, Sweden; 3 University Health Care Research Center, Faculty of Medicine and Health, Örebro University, Örebro, Sweden

**Keywords:** Weight loss support, Health problems, Prevalence, Population studies

## Abstract

**Objective:**

The aim of this study was to investigate factors associated with reported need of weight loss support among adults with overweight or obesity in the general population.

**Design:**

A cross-sectional population study based on a survey questionnaire sent to a random population sample. Multivariate odds ratios for reported need of weight loss support were calculated for socio-economic, lifestyle and health indicators, in total and by gender and age group.

**Setting:**

Five counties in Sweden in 2022.

**Participants:**

The study includes 10 069 persons with overweight or obesity (BMI ≥ 25 kg/m^2^) aged 30–69 years. BMI was based on self-reported weight and height.

**Results:**

In total, about 20 % reported needing weight loss support. The factors most strongly associated with reported need of weight loss support were obesity and female gender. Lack of social support, economic difficulties, physical inactivity, poor self-rated health, musculoskeletal pain and depression were also associated with reported need of weight loss support, whereas diabetes and hypertension were not. Some differences in these associations were observed between age groups.

**Conclusion:**

Reported need of weight loss support is more common among women than among men and associated with obesity, lack of social support, economic difficulties, physical inactivity, poor self-rated health, musculoskeletal pain and depression in both genders. These factors are important for planning preventive and weight-control measures among adults with overweight or obesity.

The prevalence of obesity is a major public health problem worldwide^(1,2)^ that has continued to increase in many countries during the COVID-19 pandemic^(3,4)^. The causes of obesity are complex including biological, genetic, environmental, economic, social and psychological factors^(5,6)^. The chronic nature of obesity is associated with multiple health complications and increased risk of disability as well as increased healthcare costs^(7–9)^. Evidence-based methods to lose weight are therefore important to tackle the increasing obesity trends in the adult population, but the long-term outcomes of weight loss attempts are discouraging^(10,11)^.

Previous studies show that the prevalence of weight control attempts in the adult population is high, about 40–50 %^(12,13)^. In addition, a study from England found that an increase has occurred in this proportion in recent decades^(14)^. The main determinants of weight control attempts are high BMI and female gender^(12–14)^. Women try to lose weight to a greater extent than men, although overweight is more common among men. The proportion trying to lose weight decreases with higher age and weight control attempts are more common among persons with high socio-economic status^(12,15)^.

Although the problems of overweight and obesity are difficult to manage without professional help, previous studies indicate that most individuals, who are ready to lose weight, try to do this on their own^(16,17)^. In an international study, 81 % of people with obesity assumed full responsibility for weight loss, but a majority reported that they would like a healthcare professional to initiate a conversation about weight management^(18)^.

A population study in the Netherlands in 2012 found that the factors most strongly associated with needing help for weight loss were obesity and poor perceived health among men and obesity and young age among women^(17)^. Thus, they reported differences between genders and age groups in these associations. In a previous population-based study carried out in Sweden in 2017, we studied the proportion of people in the general adult population who wanted to lose weight and found that persons with overweight or obesity who wanted to lose weight and needed weight loss support are a special group with higher frequency of poor self-rated health and different types of health problems, including mental health problems^(19)^.

In the present study, the aims were to study (1) the proportion of persons with overweight or obesity in the general population who report needing weight loss support, this time after the COVID-19 pandemic, (2) to examine which factors are independently associated with reported need of weight loss support and (3) to investigate whether there are differences in these associations by gender and age group.

## Methods

The study is based on a survey questionnaire sent to a random population sample in five counties in Sweden in February–May 2022. The questionnaire comprised questions about lifestyle habits, living conditions and health and included questions about desire for weight loss and need of weight loss support. The sampling frame was the population register at Statistics Sweden, the statistical administrative authority in Sweden, covering all inhabitants of the study area. The area investigated covers five counties (Sörmland, Uppsala, Värmland, Västmanland and Örebro) including fifty-five municipalities with over one million inhabitants in the central part of Sweden. The questionnaire was sent to 78 117 persons in the age group 18 years or older, corresponding to about 6 % of the population. The random sample was stratified by gender, age group and municipality to allow to monitor living conditions, lifestyle factors and health in different groups of the population. The questionnaire was sent by mail and could be answered by mail or online. Data collection was discontinued after two postal reminders. Up to three short message service reminders were also sent to persons with a registered mobile phone number. In total, 35 169 persons aged 18 years or older answered the questionnaire. The overall response rate was 45 %. The current study included a subsample of 10 069 persons aged 30–69 years with overweight or obesity (BMI ≥ 25 kg/m^2^) who had answered the question about desire for weight loss.

### Outcome

In the questionnaire, the respondent was asked if he/she wanted to change his/her weight with the answer options: ‘No’, ‘Yes, I want to lose weight’, ‘Yes, I want to gain weight’. This question is a modified version of the question used in the study by Hjärtåker et al^(15)^. A supplementary question to those who wanted to change their weight was: ‘If you want to change your weight, do you think you can manage it yourself?’ (with the answer options ‘Yes’ and ‘No, I need support’).

### Socioeconomic factors

Economic difficulties were estimated with the question ‘During the last 12 months, have you ever had difficulty in managing the regular expenses for food, rent, bills etc.?’ (‘No’, ‘Yes, once’, ‘Yes, more than once’). The alternatives were dichotomised into no and yes.

Social support was measured with the question: ‘Do you have anyone you can share your innermost feelings with and confide in?’ (yes/no).

### Lifestyle factors

Two questions for measuring physical activity, similar to those in the Swedish national public health survey^(20)^, were used. The first question was ‘How much time do you spend in a normal week on physical training that leaves you out of breath – for example running, fitness training or ball sports?’ The response options were 0 min/no time; less than 30 min; 30–59 min (0·5–1 h); 60–89 min (1–1·5 h); 90–119 min (1·5–2 h); 2 h or more. The second question was ‘How much time do you spend in a normal week on daily activities – for example walking, cycling or gardening? Count all time together.’ The response options were: 0 min/no time; less than 30 min; 30–59 min (0·5–1 h); 60–89 min (1–1·5 h); 90–149 min (1·5–2·5 h); 150–299 min (2·5–5 h); 5 h or more. These questions are used to measure whether the respondent reaches 150 activity minutes of moderate-to-vigorous physical activity per week as recommended by the WHO^(21)^. The minutes spent in physical training (first question) are doubled when the sum of the minutes from the two questions is calculated^(20)^.

### Health indicators

BMI was based on self-reported weight and height and was calculated as weight in kilograms divided by height in metres squared. Overweight was defined according to the WHO as BMI ≥ 25 kg/m^2^ and obesity as BMI ≥ 30 kg/m^2(22)^.

Obesity is associated with several diseases and symptoms, such as hypertension, diabetes, musculoskeletal pain and depression^(6,9,23)^. Therefore, we investigated the association between several health problems and reported need of weight loss support. Diagnosed diseases were measured with the following question: ‘Do you have any of the following diagnosed illnesses?’ (with answer options yes/no). Questions about diseases included diabetes, high blood pressure and depression.

Musculoskeletal pain was derived from the question: ‘Do you have any of the following discomforts or symptoms?’ where one of the symptoms was ‘aches in your shoulders or neck’. The answer categories were ‘No’, ‘Yes, minor discomfort’ and ‘Yes, severe discomfort’, where the latter two categories were combined to Yes.

Self-rated health was also included in the analyses and measured on a five-grade scale with the options ‘very good’, ‘good’, ‘fair’, ‘poor’ and ‘very poor’. The last two options were classified as poor self-rated health.

### Ethical considerations

The data on gender, age and educational level are based on registry data from Statistics Sweden. By answering the questionnaire, participants gave their informed consent that questionnaire data would be linked to Swedish official registers at Statistics Sweden through personal identification numbers. After the record linkage, all identity information was removed before the material was handed over from Statistics Sweden to the regions for further processing. Authorisation from the Swedish Ethical Review Authority has been obtained (Dnr 2021–05814–01). The data material is protected according to the Public and Privacy Act (2009: 400, Chapter 24, Section 8).

### Statistical analyses

Subjects were divided into two groups: persons with overweight or obesity who do not want to lose weight or who want to lose weight but do not need support (*n* 8064) and those who want to lose weight and need support (*n* 1951). *χ* ^2^ tests were used to test the statistical significance of the differences between groups in the proportion needing weight loss support. Multivariate logistic regression analyses were conducted to assess variables independently associated with needing weight loss support. These variables included gender, age group, economic difficulties, social support, physical activity, obesity, self-rated health and the included health problems. The results from the logistic regression analyses are presented as adjusted OR with 95 % CI. In total, 9755 subjects with non-missing values for the independent variables were included in the multivariate logistic regression analysis for the total group. The multivariate analyses were also run separately for men and women and by 10-year age groups. All analyses were carried out using SPSS, version 28.0.

Since those who do not want to lose weight may differ from those who want to lose weight but do not need weight loss support, we conducted a supplementary analysis and excluded those who do not want to lose weight (*n* 1581) from the multivariate logistic regression model. In the supplementary analysis, 8234 subjects, who wanted to lose weight and had non-missing data for variables included in the model, were included in the same model as used in the original analysis.

## Results

In total, 76 % of the subjects with overweight and 93 % of the subjects with obesity reported that they wanted to lose weight. The proportion who wanted to lose weight was higher among women (89 %) than among men (76 %). It was also higher among those with high (86 %) and middle (82 %) educational levels compared with those with low educational level (72 %).

About 20 % of the adults with overweight or obesity reported needing weight loss support (Table 1). No large differences were observed between educational levels, but those with obesity (35 %) reported much more often needing weight loss support than those with overweight (10 %). Reported need of weight loss support was also more common among women (26 %) than among men (13 %). Differences between age groups were statistically significant but rather small.

**Table 1.** Background characteristics of the study population and proportion who reported that they need weight loss support among persons with overweight or obesity, 30–69 years (Numbers and percentages)

	Total		Needs weight loss support	*P* value for difference in % needing weight loss support
	*n*	%	%	
Total	10 069	100	19·5	
Gender				< 0·001
Woman	5092	50·6	25·8	
Man	4977	49·4	13·1	
Age				< 0·001
30–39 years	1457	14·5	20·1	
40–49 years	2085	20·7	22·0	
50–59 years	2982	29·6	19·7	
60–69 years	3545	35·2	17·7	
Educational level				< 0·590
Low	1036	10·3	20·7	
Medium	5105	51·0	19·3	
High	3874	38·7	19·4	
BMI category				< 0·001
Overweight	6265	62·2	9·9	
Obesity	3804	37·8	35·3	

In the multivariate analysis, the factors most strongly associated with reported need of weight loss support were obesity (OR: 4·2; 95 % CI: 3·8, 4·7), female gender (OR: 2·2; 95 % CI: 2·0, 2·5), lack of social support (OR: 1·9; 95 % CI: 1·6, 2·2) and poor self-rated health (OR: 1·8; 95 % CI: 1·5, 2·2) (Table 2). Economic difficulties, physical inactivity, musculoskeletal pain and depression were also statistically significantly associated with reported need of weight loss support. Diabetes and hypertension had no independent association with reported need of weight loss support. Educational level was not included in the multivariate analysis since it was not associated with needing weight loss support. Obesity, lack of social support and poor self-rated health had the strongest associations also when analysed separately in men and women. The age group 40–49 years had the highest prevalence of reported need of weight loss support among men, whereas no differences between age groups were found among women.

**Table 2.** Proportion who reported that they need weight loss support by variable category and adjusted OR (with 95 % confidence intervals in parentheses) for reported need of weight loss support among persons with overweight or obesity, 30–69 years (Odds ratios and 95 % confidence intervals)

				Gender				Age group							
		Total		Men		Women		30–39 years		40–49 years		50–59 years		60–69 years	
	%	OR*	95 % CI	OR*	95 % CI	OR*	95 % CI	OR*	95 % CI	OR*	95 % CI	OR*	95 % CI	OR*	95 % CI
Gender															
Men	13·1	1		–		–		1		1		1		1	
Women	25·8	**2·2**	**2·0, 2·5**	–		–		**2·3**	**1·7, 3·2**	**1·7**	**1·4, 2·2**	**2·7**	**2·2, 3·3**	**2·2**	**1·8, 2·7**
Age															
30–39 years	20·1	1		1		1		–		–		–		–	
40–49 years	22·0	1·2	1·0, 1·4	**1·4**	**1·0, 2·0**	1·1	0·9, 1·3	–		–		–		–	
50–59 years	19·7	1·1	0·9, 1·3	1·0	0·7, 1·3	1·1	0·9, 1·4	–		–		–		–	
60–69 years	17·7	1·0	0·9, 1·2	1·0	0·7, 1·4	1·0	0·8, 1·3	–		–		–		–	
BMI category															
Overweight	9·9	1		1		1		1		1		1		1	
Obesity	35·3	**4·2**	**3·8, 4·7**	**4·9**	**4·0, 5·9**	**3·9**	**3·4, 4·5**	**4·4**	**3·3, 6·0**	**3·7**	**2·9, 4·7**	**4·6**	**3·7, 5·7**	**4·3**	**3·5, 5·2**
Economic difficulties															
No	17·4	1		1		1		1		1		1		1	
Yes	34·2	**1·5**	**1·3, 1·8**	**1·7**	**1·3, 2·2**	**1·4**	**1·2, 1·7**	**1·9**	**1·3, 2·6**	**1·7**	**1·3, 2·2**	**1·5**	**1·1, 2·0**	1·1	0·8, 1·6
Social support															
Yes	17·7	1		1		1		1		1		1		1	
No	32·0	**1·9**	**1·6, 2·2**	**2·0**	**1·6, 2·5**	**1·7**	**1·4, 2·1**	**2·4**	**1·6, 3·5**	**2·0**	**1·5, 2·7**	**1·8**	**1·3, 2·4**	**1·7**	**1·3, 2·2**
Physical activity															
Yes	15·0	1		1		1		1		1		1		1	
No	27·2	**1·5**	**1·4, 1·7**	**1·7**	**1·4, 2·1**	**1·4**	**1·2, 1·6**	1·3	1·0, 1·8	**1·6**	**1·2, 2·0**	**1·8**	**1·5, 2·2**	**1·4**	**1·2, 1·7**
Self-rated health															
Good or fair	4·8	1		1		1		1		1		1		1	
Poor	15·3	**1·8**	**1·5, 2·2**	**2·1**	**1·5, 2·9**	**1·7**	**1·3, 2·1**	**2·2**	**1·3, 3·6**	**1·7**	**1·1, 2·5**	**1·8**	**1·2, 2·5**	**1·9**	**1·3, 2·7**
Diabetes															
No	19·0	1		1		1		1		1		1		1	
Yes	24·5	0·9	0·8, 1·1	1·0	0·8, 1·4	0·9	0·7, 1·1	0·5	0·2, 1·4	1·1	0·7, 1·9	1·0	0·7, 1·5	0·9	0·7, 1·2
Hypertension															
No	18·0	1		1		1		1		1		1		1	
Yes	22·7	1·1	0·9, 1·2	1·2	0·9, 1·4	1·0	0·9, 1·2	0·7	0·4, 1·2	1·3	1·0, 1·8	1·1	0·9, 1·3	1·0	0·8, 1·2
Depression															
No	17·6	1		1		1		1		1		1		1	
Yes	37·5	**1·4**	**1·2, 1·7**	**1·7**	**1·2, 2·3**	**1·3**	**1·1, 1·6**	1·4	1·0, 2·1	1·2	0·9, 1·7	**1·4**	**1·0, 1·9**	**1·7**	**1·2, 2·3**
Pain in shoulders or neck															
No	13·5	1		1		1		1		1		1		1	
Yes	24·2	**1·5**	**1·3, 1·7**	**1·5**	**1·3, 1·8**	**1·5**	**1·3, 1·8**	**1·5**	**1·1, 2·0**	**1·7**	**1·3, 2·1**	**1·5**	**1·2, 1·9**	**1·5**	**1·2, 1·8**

* Adjusted OR from multivariate logistic regression models simultaneously adjusted for all the independent variables listed in the table. Statistically significant OR are marked with bold.

When the associations were examined separately in the 10-year age groups, many of the associations were similar to those in the total group, but some differences were observed (Table 2). Physical inactivity was not associated with reported need of weight loss support in the youngest age group 30–39 years, while the association between economic difficulties and reported need of weight loss support was not statistically significant in the oldest age group 60–69 years. Depression was not associated with reported need of weight loss support in the age groups 30–39 and 40–49 years. Lack of social support, poor self-rated health and musculoskeletal pain were associated with reported need of weight loss support in all age groups, whereas obesity had the strongest association in all age groups with ORs between 3·7 and 4·6.

When those who do not want to lose weight were excluded from the multivariate analysis, similar odds ratios were found as in the original model for the total study population (not shown). Obesity, female gender, lack of social support and poor self-rated health were most strongly associated with reported need of weight loss support, whereas economic difficulties, physical inactivity, musculoskeletal pain and depression also showed statistically significant OR.

## Discussion

In this population-based study carried out in Sweden in 2022 among men and women aged 30–69 years with overweight or obesity, about one in five reported needing support to lose weight. This proportion increased with higher BMI: 10 % of the persons with overweight and 35 % of those with obesity reported that they need support to lose weight. Other factors strongly associated with reported need of weight loss support were female gender, lack of social support and poor self-rated health.

The proportion who wanted to lose weight was 76 % among persons with overweight and 93 % among persons with obesity in the current study, which is in line with the corresponding survey carried out in 2017, i.e. before the COVID-19 pandemic^(19)^. These results can be compared with an Australian study among primary care patients, where 66 % of the patients with overweight and 86 % of the patients with obesity intended to lose weight in the next 6 months^(24)^. Health reasons were the main motivating factors for attempting weight loss among persons with overweight or obesity in that study^(24)^. A population-based study in Switzerland carried out among women with overweight or obesity in the age group 40–59 years found that 90 % wanted to lose weight^(25)^. This aligns with our results where 89 % of the women with overweight or obesity aged 30–69 years wanted to lose weight.

The proportion who reported needing weight loss support was slightly higher in 2022 (20 %) than the corresponding proportion in 2017 (18 %). The proportion was higher among women (26 %) than among men (13 %). The corresponding proportion in the 2017 survey was 22 % in women and 12 % in men, suggesting a slightly higher proportion in the current study in women while the proportion in men was about the same. A population study in the Netherlands in 2012 found a somewhat higher proportion of need for help for weight loss (25 %), but with a corresponding difference between women (32 %) and men (19 %)^(17)^. One reason for the higher proportion in the Dutch study may be that the age group studied was somewhat younger, 19–64 years, with the highest prevalence among the youngest women. Other reasons may be that the questions were not exactly the same and that the Dutch study was carried out in 2012. The lower reported need of weight loss support among men may be due to that men are less likely to recognise their weight as a health risk compared to women^(26)^, that men put lower priority to health behaviour^(27)^ or that men experience weight management services as female-dominated and designed for women^(28)^.

The factors most strongly associated with reported need of weight loss support were obesity and female gender, in line with the Dutch study^(17)^. In the current study, poor self-rated health was associated with reported need of weight loss support in both men and women, whereas the Dutch study found a similar association in men but not in women. Musculoskeletal pain and depression were also associated with reported need of weight loss support among both genders. However, diabetes and hypertension were not independently associated with reported need of weight loss support. In the Dutch study^(17)^, comorbidity was associated with reported need of weight loss support in men but not in women, but specific diagnoses were not studied separately. In the present study, the age group 40–49 years had the highest prevalence of reported need of weight loss support in men, while no differences between age groups were found in women.

In our study, there was a statistically significant association between physical inactivity and reported need of weight loss support in the age group 40–69 years, while the Dutch study found no such association^(17)^. The results in our study indicate that middle-aged persons who report that they need support to lose weight are more physically inactive than persons who do not report needing weight loss support. Increased physical activity may therefore be one way to support weight loss among middle-aged persons. In addition, there was an independent association between economic difficulties and reported need of weight loss support in the present study, except in the oldest age group 60–69 years. This suggests that in the age group 30–59 years, economic difficulties may be an obstacle for trying to lose weight.

Lack of social support was associated with reported need of weight loss support in the current study. Obesity is commonly accompanied with stigma^(29)^. Weight-related stigma has been recognised as a contributor to negative health outcomes as well as behaviours that can promote and exacerbate obesity and should therefore be considered an important aspect of obesity prevention and treatment^(30)^. This underlines the importance of social support when designing measures for weight loss management.

Depression was one of the diagnoses related to reported need of weight loss support in the current study, although this association was statistically significant only in the oldest age groups 50–69 years. Previous studies have shown that obesity is associated with depression^(31)^, although this association may vary by age group^(32)^. There is also a bi-directional relationship with depression also leading to higher risk of obesity^(23,31)^. Poor health behaviours, comorbidities and side effects of antidepressant medications have been suggested as plausible explanations for the association between depression and an increased risk of obesity^(31)^. In addition, weight history, such as having had more weight loss attempts and a greater weight loss being desired, has been shown to be associated with symptoms of depression^(33)^. The higher prevalence of depression among those needing weight loss support should therefore be taken into account when weight loss management interventions are designed.

Evidence-based weight loss methods are important to tackle the globally increasing trends of obesity in the adult population^(1–4)^. However, the long-term results of weight loss attempts are discouraging^(10,11)^. In addition, the weight management services provided by the healthcare systems seem not very effective. The international ACTION-IO study, carried out in 11 countries, found that of the 14 502 included persons with obesity, 11 % had at least 5 % weight loss and 5 % had at least 10 % weight loss over the last 3 years that was maintained for 1 year or more^(18)^. This indicates that maintenance of large weight loss is rare. In the National Health and Nutrition Examination Survey (NHANES) in the USA, 40 % of the persons with overweight or obesity received weight counselling from healthcare providers and about half of those advised to make changes reported doing so^(34)^. Therefore, the study recommended that the weight management services should be tailored to the social contexts of populations less likely to adopt weight control recommendations. In a UK study, only 20 % of a large sample of patients with obesity had sought GP support for weight loss in the last 12 months; instead, most efforts to lose weight were self-guided and did not use evidence-based strategies^(35)^. In a recent population-based study^(36)^, a contact with a primary health care centre for support regarding lifestyle changes was not the first choice for most adults living in Sweden. The authors concluded that to adequately work with health-promotive and disease-preventive strategies at primary health care centres, these settings need to find valid methods for engaging with and meeting the needs of a population struggling with unhealthy lifestyles^(36)^.

In our study, the group that reported needing weight loss support had many different types of health problems including musculoskeletal pain, depression and poor self-rated health. This is in line with the previous studies underlining the importance of designing weight-control measures that meet the social context of the affected population^(34,36)^. In the current study, it was not specified whether the reported need of weight loss support meant support from the healthcare provider or, for example, from family or friends. However, because of the high prevalence of various types of health problems, it is important that the support should be provided by the healthcare system. The finding that lack of social support, economic difficulties, physical inactivity, poor self-rated health, musculoskeletal pain and depression were associated with reported need of weight loss support underscores that the group needing weight loss support is more affected by social factors and health problems than other groups. The supplementary analysis confirmed that this is true irrespective of whether those who reported needing weight loss support are analysed in relation all other subjects with overweight or obesity or in relation to subjects with overweight or obesity who want to lose weight. Future studies should investigate what type of weight loss support would be most suited for the group needing weight loss support.

The results of the current study, based on data gathered in 2022, confirm some of the results of the corresponding study in 2017, e.g. the association between reported need of weight loss support and several health problems^(19)^. However, in the current study, also independent relationships were studied, i.e. taking into account all included socio-economic, lifestyle and health indicators simultaneously. In addition, differences, in the associations between these factors and reported need of weight loss support, between men and women and between age groups were studied in the current study.

### Strengths and limitations of the study

There are several limitations with the current study. The study was cross-sectional in design which precludes any interpretations about causality. However, the purpose of the study was to describe the group reporting need of weight loss support and therefore a cross-sectional study design is not a major problem. The response rate of the survey was 45 %, which may affect the representativeness of the respondents^(37)^. In addition, BMI was based on self-reported weight and height, which may lead to an underrepresentation of persons with overweight or obesity in the study population^(38)^. Another limitation is with regard to the dichotomisation of the independent variables, which might reduce the specificity of the data.

One of the advantages of the present study is that it is based on a considerable random sample of the general population in a large geographical area, whereas many of the previous studies have been based on healthcare patients. It also represents a large sample of men and women with overweight or obesity in the age range 30–69 years. In addition, a variety of socioeconomic, lifestyle and health indicators in relation to reported need of weight loss support could be assessed. There are few previous population-based studies on this research topic, and since the present study was conducted in 2022, it provides a current picture of the need for weight loss support in the general population.

### Conclusion

In this large population-based study, carried out in Sweden in 2022 among men and women aged 30–69 years with overweight or obesity, reported need of weight loss support was more common among women than among men and strongly associated with obesity in both genders. In addition, lack of social support, economic difficulties, physical inactivity, poor self-rated health, musculoskeletal pain and depression were associated with reported need of weight loss support. These factors are important to consider when planning preventive and weight management interventions for adults with overweight or obesity.
